# Renal Ultrasonography Screening: A Comparison Between Male and Female Cohorts of University Students

**DOI:** 10.7759/cureus.101831

**Published:** 2026-01-19

**Authors:** Mahmoud Babiker, Ghadi M Almehmadi, Faye Al-Masoud, Sara H Basrawi

**Affiliations:** 1 Diagnostic Radiology Technology, College of Applied Medical Sciences, Taibah University, Madinah, SAU

**Keywords:** body mass index, gender comparison, renal screening, ultrasonography, university students

## Abstract

Objective: This study aimed to assess renal ultrasonography findings in a cohort of university students.

Materials and methods: Participants underwent renal ultrasonography screening in a prospective cross-sectional study using the Esaote MyLab40 system (Genoa, Italy). A 3.5-MHz transducer was used to evaluate the right (Rt) and left (Lt) kidneys for parenchymal texture and length measurements. Participants were selected using simple random sampling. Inclusion criteria consisted of asymptomatic university students. Descriptive statistics, t-tests, and Pearson correlation analyses were performed.

Results: A total of 100 participants with a mean age of 20.91 ± 1.52 years were enrolled. The mean kidney length was 9.68 ± 0.73 cm on the Rt side and 10.02 ± 1.19 cm on the Lt side. The mean body mass index (BMI) was 23.45 ± 5.24 kg/m². Incidental renal findings were observed in 5% of participants and were more frequent in males, including simple cysts (2%), renal agenesis (1%), and a double collecting system (2%). For males and females, respectively, the mean Rt kidney length was 9.9630 ± 0.56225 cm and 9.3904 ± 0.78268 cm, while the mean Lt kidney length was 9.9424 ± 1.58818 cm and 10.1046 ± 0.58872 cm. Females demonstrated slightly longer kidney lengths overall (t = 4.201, *P* < 0.001), although no significant difference was observed for the Rt kidney alone. A significant correlation was noted between Rt kidney length and BMI (r = 0.293, p = 0.003).

Conclusion: Incidental renal sonographic findings were uncommon and occurred more frequently in males. Females demonstrated slightly longer kidney lengths. Kidney length showed a significant correlation with BMI.

## Introduction

Preservation of kidney health is an important clinical concern, as the kidneys perform several essential functions, including blood filtration, metabolism of waste products, and excretion of excess ions while retaining vital substances in the bloodstream. The kidneys also play a key role in regulating blood pressure and maintaining electrolyte balance [1]. The normal average length of an adult kidney may reach approximately 12 cm [2]. Renal size is therefore a valuable parameter for the diagnosis of renal function disorders and for guiding related clinical management [3].

Several clinical laboratory tests are used to evaluate kidney function. The most practical tests include quantification of proteinuria (albuminuria) and estimation of the glomerular filtration rate [4]. Ultrasonography is considered the ideal initial imaging modality for patients with abnormal kidney function [5]. Previous studies [6] have suggested that ultrasonography is an effective tool for detecting asymptomatic renal malignancies and have highlighted the need to optimize screening guidelines to reduce costs. Renal ultrasonography screening has a reported sensitivity of 82%-83% and specificity of 98%-99% [7]. In addition, ultrasonography allows assessment of both the structural and functional aspects of renal patency [5].

The current study involves real-time renal sonographic screening of a university student population as part of the university’s free healthcare services. This approach may aid in the early detection and prediction of renal abnormalities. The objective of this study was to assess renal findings in a cohort of university students using ultrasonography. Additionally, the study aimed to contribute to early preventive healthcare by identifying renal abnormalities in young individuals, to evaluate the correlation between renal length and body mass index (BMI), and to compare renal length between male and female participants.

The research questions were: Are there significant ultrasonographic renal findings among university students, and is there a correlation between renal length and participants’ BMI?

## Materials and methods

A descriptive cross-sectional study was conducted from November 2024 to February 2025 at the Diagnostic Radiology Department of Taibah University. One hundred university students from the College of Applied Medical Sciences and the College of Nursing were included (50 males and 50 females). All participants underwent renal ultrasonography screening using the Esaote MyLab40 system (Genoa, Italy) with a 3.5-MHz transducer. The study included a homogeneous sample of asymptomatic Taibah University students. Students with known renal disease and those unwilling to participate were excluded. No additional eligibility criteria were applied other than current enrollment as a university student. Participants were selected using a simple random sampling technique, with each student having an equal chance of selection. This study hypothesized that incidental renal findings could be detected and that a statistically significant correlation exists between renal length and BMI among this cohort of university students.

Data collection

**Figure 1:**
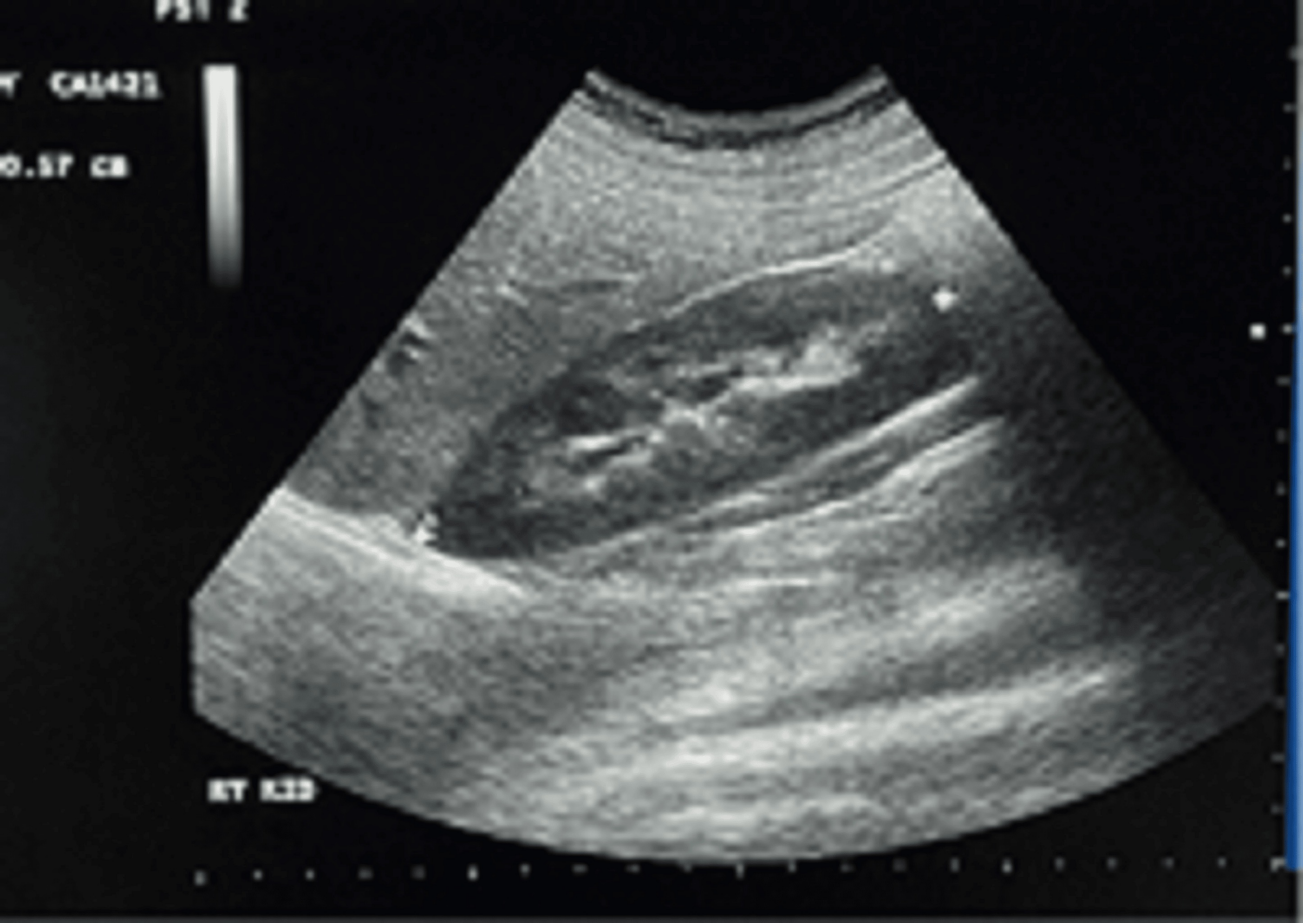
Longitudinal scan of a right kidney showing the renal length measurement method.

A data collection sheet was used to organize and record the study data. The study variables included participants’ age, weight, and height, as well as ultrasonographic findings such as kidney length, parenchymal texture, and other relevant features. A convex probe was used to obtain high-resolution images of both kidneys. Ultrasound examinations were performed with participants positioned on the examination table in the supine and lateral decubitus positions (supine for the right (Rt) kidney and lateral decubitus for the left (Lt) kidney. The probe was placed subcostally with gentle pressure to optimize kidney visualization. The liver (for the Rt kidney) and the spleen (for the Lt kidney) were used as acoustic windows, when necessary, to enhance image clarity. Both Rt and Lt renal lengths were measured in the longitudinal plane from the superior to the inferior renal pole (Figure 1). Standard gain, depth, and focus settings were adjusted as needed to optimize image quality. Renal lengths of both kidneys were measured for all participants and correlated with BMI. BMI was calculated using the formula: BMI = weight (kg)/height (m²) [8]. Ultrasound examinations were performed by two experienced sonologists, each with more than 10 years of expertise in abdominal ultrasonography, using the same standardized technique described above. Each participant underwent a single renal ultrasound examination, with kidney measurements recorded. Male participants were scanned by a male sonologist, and female participants were scanned by a female sonologist.

Ethical considerations

Ethical approval was obtained from the Deanship of Postgraduate Studies and Scientific Research (Approval No. 2025/204/304 RAD). No identifiable participant data were collected. Written informed consent was obtained from all participants prior to the ultrasound examination.

Statistical analysis

Data were analyzed using Microsoft Excel (2021) (Microsoft Corp., Redmond, WA, USA) and the IBM SPSS Statistics for Windows, Version 26.0 (Released 2018; IBM Corp., Armonk, NY, USA). Categorical and continuous variables were summarized using frequencies, percentages, means, and standard deviations. Scatterplots and histograms were used to assess data normality. Independent t-tests were performed to compare renal lengths between male and female participants, and Pearson’s correlation analyses were conducted to evaluate the relationship between renal length and BMI. A p-value < 0.05 was considered statistically significant.

## Results

Descriptive statistics

The study included 100 participants. The mean age was 20.91 ± 1.52 years (range: 18-27). The mean length of the Rt kidney was 9.68 ± 0.73 cm, and the mean length of the Lt kidney was 10.02 ± 1.19 cm. The mean BMI was 23.45 ± 5.24 kg/m² (range: 14.4-41.4) (Table 1).

**Table 1:** Descriptive statistics of the study participants.

Variables	Minimum	Maximum	Mean	Std. deviation
Age	18	27	20.91	1.518
Right kidney length	8.00	11.40	9.6767	0.73652
Left kidney length	0.00	11.68	10.0235	1.19441
Body mass index	14.4	41.4	23.448	5.2420

Ultrasound findings

**Figure 2:**
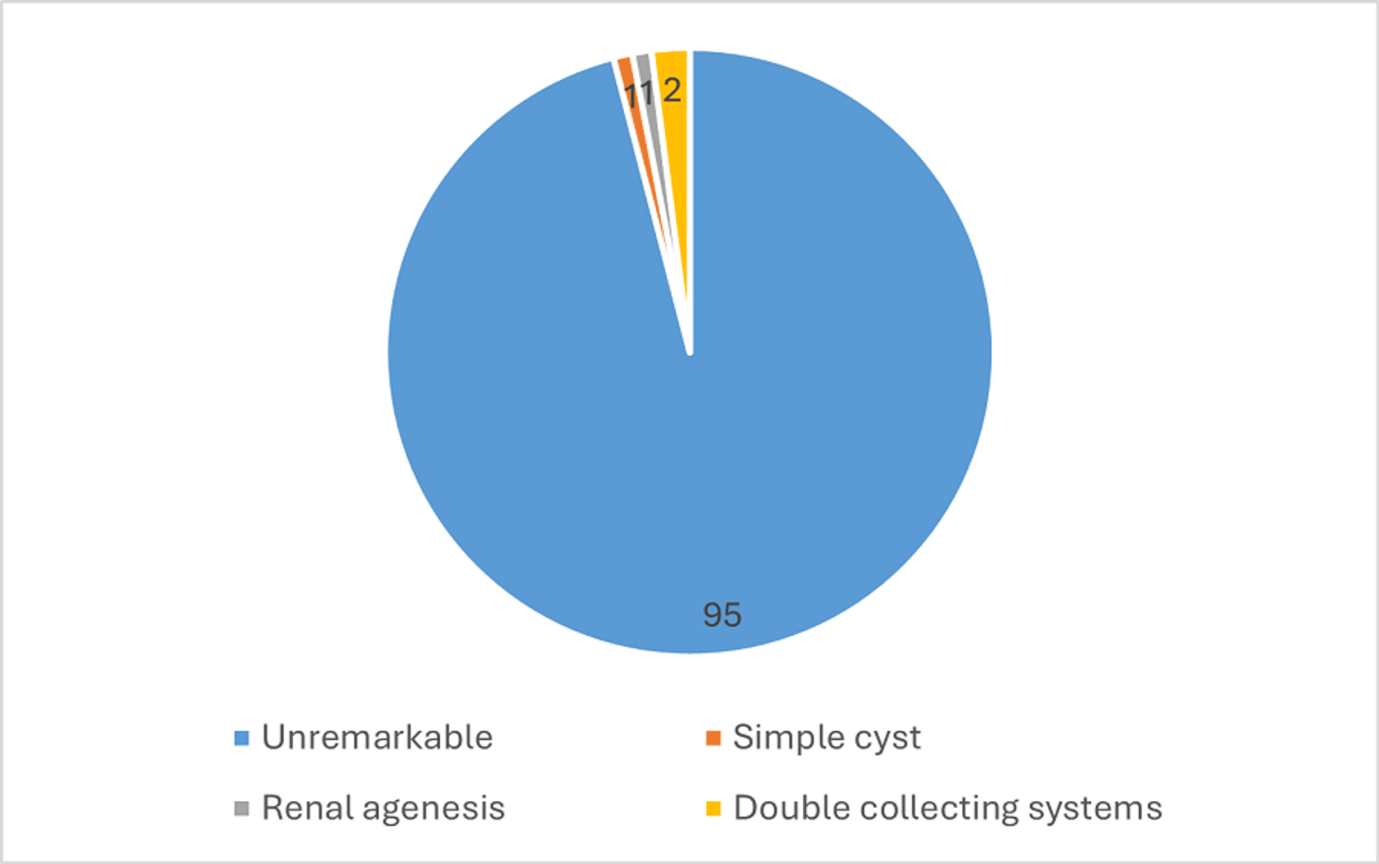
Renal ultrasound findings among the study participants.

Figure 2 summarizes the renal ultrasonography findings. The majority of participants (95%) had unremarkable kidneys, while 5% demonstrated abnormalities. These included simple cysts (2%), Lt renal agenesis (1%), and a double collecting system (2%). All abnormal findings were observed in male participants.

Statistical tests

Scatterplot and histogram analyses indicated a normal distribution of the study data. Table 2 presents the results of independent t-tests comparing the Rt kidney length between genders. A statistically significant difference was observed (t = 4.201, p < 0.001), with females having longer Rt kidneys than males (mean ± SD: 9.96 ± 0.56 cm vs. 9.39 ± 0.78 cm, respectively).

**Table 2:** Independent t-test results comparing the right kidney length between genders.

Gender	N	Mean	Std. deviation	t	p-value	Statistical significance
Male	50	9.3904	0.78268	4.201	0.000	Significant
Female	50	9.9630	0.56225			

Table 3 presents the results of independent t-tests comparing the Lt kidney length between genders. No statistically significant difference was observed (t = 0.677, p = 0.500). However, females had slightly longer Lt kidneys than males (mean ± SD: 10.10 ± 0.59 cm vs. 9.94 ± 1.59 cm, respectively).

**Table 3:** Independent t-test results comparing the left kidney length between genders.

Gender	N	Mean	Std. deviation	t	p-value	Statistical significance
Male	50	9.9424	1.58818	0.677	0.500	Insignificant
Female	50	10.1046	0.58872			

Table 4 and Table 5 present Pearson correlation coefficients between kidney length and BMI. The analysis revealed a statistically significant positive correlation between the Rt kidney length and BMI (r = 0.293, p = 0.003). In contrast, no significant correlation was observed between Lt kidney length and BMI (r = 0.177, p = 0.077).

**Table 4:** Pearson correlations between the right kidney length and body mass index of the study participants.

		Right kidney length	Body mass index
Right kidney length	Pearson correlation	1	0.293
	Sig. (2-tailed)		0.003
	N	100	100
Body mass index	Pearson correlation	0.293	1
	Sig. (2-tailed)	0.003	
	N	100	100

**Table 5:** Pearson correlations between the left kidney length and body mass index of the study participants.

		Left kidney length	Body mass index
Left kidney length	Pearson correlation	1	0.177
	Sig. (2-tailed)		0.077
	N	100	100
Body mass index	Pearson correlation	0.177	1
	Sig. (2-tailed)	0.077	
	N	100	100

## Discussion

A recent study of renal ultrasonography screening among university students found that 95% of the 100 participants had unremarkable ultrasound findings. A small number of participants had minor abnormalities, including simple cysts, renal agenesis, and a double collecting system (Figure 2). Overall, the mean Rt kidney length was 9.68 ± 0.73 cm, the mean Lt kidney length was 10.02 ± 1.19 cm, and the mean BMI was 23.45 ± 5.24 (Table 1).

Incidental renal abnormalities were present in 5% of participants (Figure 2). Among these, simple cysts accounted for 2%, while renal agenesis and a duplicated collecting system were each observed in 1%-2% of participants. These findings align with previous studies by Mazziotti et al. [9] and Di Vece et al. [10], who reported that most incidentally detected cystic lesions on ultrasound are simple cysts. Both studies also emphasized that additional imaging is sometimes necessary to distinguish benign from malignant renal masses. Similarly, Alen et al. [11] reported that incidental renal cysts are common, with a prevalence of approximately 6.5% in their study population.

In the present study, all incidental renal findings were observed in male participants. This observation is supported by Rosenblum and Salomon [12], who reported that over 100 syndromes are associated with renal and urinary tract malformations, including bilateral renal agenesis, which occurs in 1 in 3,000 to 1 in 10,000 births and predominantly affects males. Devlieger et al. [13] also reported a higher prevalence of renal agenesis in males. Additionally, the literature indicates that the incidence of renal cysts increases with age, may be inherited or acquired, and is approximately twice as common in men as in women [14].

Regarding kidney length, the current study found that the Lt kidney was slightly larger than the Rt kidney, with mean lengths of 10.02 ± 1.19 cm and 9.68 ± 0.73 cm, respectively (Table 1). These results are consistent with findings by Alyami et al. [15], who reported mean Rt and Lt kidney lengths of 9.7 cm and 10.1 cm, respectively.

When comparing kidney lengths by gender, the mean Rt kidney length was 9.39 cm in males and 9.96 cm in females, while the mean Lt kidney length was 9.94 cm in males and 10.10 cm in females (Tables 2, 3). Although renal lengths were generally similar between males and females, a statistically significant difference was observed for the Rt kidney. These findings partially align with Saleem et al. [16], who reported no significant difference in renal length between genders. In contrast, Karim [17] and Alyami et al. [15] reported that males tend to have larger kidneys, while Almanaa et al. [18] observed greater kidney thickness and Lt kidney width in males.

Regarding the relationship between renal length and BMI, this study found a significant positive correlation between Rt kidney length and BMI (Tables 4, 5). These results are consistent with Saleem et al. [16], who reported a significant association between renal size and demographic factors including age, sex, and BMI. Similarly, Bhardwaj et al. [19] found a significant correlation between kidney length and renal parenchymal volume with body surface area, weight, and height. However, unlike Bhardwaj et al., the present study did not find a significant correlation between renal length and gender, as females in this cohort had slightly longer kidneys on both sides.

Limitations

The main limitation of this study was its small sample size, which may limit the generalizability of the findings. In addition, the study population consisted solely of university students, resulting in limited age diversity; therefore, the results may differ in populations with a broader age range.

## Conclusions

The study successfully addressed the research question and supported the hypothesis. Incidental renal sonographic findings were rare and occurred more frequently in young men. The Lt kidney was slightly longer than the Rt, and females had slightly longer Rt kidneys than males. A significant correlation was observed between Rt kidney length and BMI in this cohort of university students. These findings highlight a potential relationship between anthropometric factors, such as height, weight, and BMI, and kidney dimensions, providing insight into how body structure may influence renal size. Clinically, renal ultrasonography screening is valuable, as participants with abnormal findings were referred for further evaluation and management. Future studies with larger sample sizes and more diverse age groups are recommended to validate and expand upon these results.
